# Tumour buds determine prognosis in resected pancreatic ductal adenocarcinoma

**DOI:** 10.1038/s41416-018-0093-y

**Published:** 2018-05-14

**Authors:** Philipp Lohneis, Marianne Sinn, Fritz Klein, Sven Bischoff, Jana K. Striefler, Lilianna Wislocka, Bruno V. Sinn, Uwe Pelzer, Helmut Oettle, Hanno Riess, Carsten Denkert, Hendrik Bläker, Anja Jühling

**Affiliations:** 1 Charité – Universitätsmedizin Berlin, corporate member of Freie Universität Berlin, Humboldt-Universität zu Berlin, and Berlin Institute of Health, Institute of Pathology, Charitéplatz 1, Berlin, 10117 Germany; 2 University Hospital Cologne, Institute of Pathology, Kerpener Strasse 62, Köln, 50924 Germany; 3 Department of Medical Oncology, CONKO study group, Haematology and Tumorimmunology, Augustenburger Platz 1, Berlin, 13353 Germany; 4 Department of Surgery Campus Charité Mitte/ Campus Virchow-Klinikum, Augustenburger Platz 1, Berlin, 13353 Germany; 5 Outpatient Department Hematology/Oncology, Friedrichstrasse 53, Friedrichshafen, 88045 Germany

**Keywords:** Pancreatic cancer, Prognostic markers, Translational research

## Abstract

**Background:**

The prognostic effect of tumour budding was retrospectively analysed in a cohort of 173 patients with resected pancreatic ductal adenocarcinomas (PDACs) of the prospective clinical multicentre CONKO-001 trial.

**Methods:**

Haematoxylin and eosin (H&E)-stained whole tissue slides were evaluated. In two independent approaches, the mean number of tumour buds was analysed according to the consensus criteria in colorectal cancer, in one 0.785 mm^2^ field of view and additionally in 10 high-power fields (HPF) (HPF = 0.238 mm^2^).

**Results:**

Tumour budding was significantly associated with a higher tumour grade (*p* < 0.001) but not with distant or lymph node metastasis. Regardless of the quantification approach, an increased number of tumour buds was significantly associated with reduced disease-free survival (DFS) and overall survival (OS) (10 HPF approach DFS: HR = 1.056 (95% CI 1.022–1.092), *p* = 0.001; OS: HR = 1.052 (95% CI 1.018–1.087), *p* = 0.002; consensus method DFS: HR = 1.037 (95% CI 1.017–1.058), *p* < 0.001; OS: HR = 1.040 (95% CI 1.019–1.061), *p* < 0.001). Recently published cut-offs for tumour budding in colorectal cancer were prognostic in PDAC as well.

**Conclusions:**

Tumour budding is prognostic in the CONKO-001 clinical cohort of patients. Further standardisation and validation in additional clinical cohorts are necessary.

## Introduction

The clinical outcome of pancreatic ductal adenocarcinoma (PDAC) is determined by the invasive spread of tumour cells in the adjacent tissue, as well as into regional lymph node. Therefore, a morphological analysis of different patterns of tumour invasion might lead to a better prognostic classification of PDAC.

Tumour budding is best described as a certain type of invasive growth pattern, often but not exclusively present at the invasion front, that is observed at histological examination of carcinomas. It is defined as isolated tumour cells or small non-glandular tumour cell cluster and commonly believed to represent epithelial–mesenchymal transition (EMT) of tumours.^1, 2^ EMT describes a process of cell-plasticity, in which cells loose epithelial and gain mesenchymal characteristics. This process promotes organ formation during embryogenesis and plays an important role during wound healing.^3^ In cancer, aberrant activation of EMT pathways promote tumour invasion, progression and metastasis formation. We studied the prognostic effect of tumour buds in a cohort of 173 PDACs from the clinical multicentre CONKO-001 trial. CONKO-001 is a prospective phase III trial, investigating the role of adjuvant gemcitabine in pancreatic cancer patients.^4^ It disposes prospectively collected clinical data and a follow-up of more than 5 years, as well as two randomised groups of patients: one treated for 6 months postoperatively with gemcitabine compared to one that was only observed.

In this translational investigation, we used H&E-stained slides of formalin-fixed paraffin-embedded tumour specimen of the CONKO-001 trial, to evaluate the hypothesis that in PDAC high numbers of tumour buds are linked to a worse prognosis. Therefore, in two independent approaches, the mean number of tumour buds were analysed, according to the recently published consensus criteria for colorectal cancer of the International Tumour Budding Consensus Conference (ITBCC),^5^ in one 0.785 mm^2^ field of view with highest tumour budding and additionally in 10 high-power fields (HPF) (HPF = 0.238 mm^2^). For both quantification methods, we examined the prognostic value of tumour budding and assessed its correlation to clinicopathological features of PDAC. Finally, we evaluated if tumour budding is associated with the presence of distant or lymph node metastasis formation in PDAC, and compared the number of tumour buds of metastasised and non-metastasised tumours of CONKO-001 with 38 PDACs with synchronous resected liver metastases.

## Methods

### CONKO-001 study-cohort

Baseline data and patient’s characteristics: Between July 1998 and December 2004, a total of 368 patients were recruited with resected pancreatic cancer (R0 and R1 resection). In an outpatient setting, adjuvant treatment with gemcitabine (1000 mg/m^2^ d1, 8, 15, q29) was continued for 6 months and compared to observation only. Median disease-free survival (DFS) (13.4 vs. 6.9 months, *p* < 0.001),^4^ overall survival (OS) (22.8 vs. 20.2 months, *p* = 0.01) and 5-year survival (20.7% vs. 10.4%)^6^ were significantly improved by gemcitabine. The study was approved by the institutional review committee (registration number EA1/139/05; trial registration isrctn.org Identifier: ISRCTN34802808).

A total of 183 formalin-fixed paraffin-embedded (FFPE) tissue samples of CONKO-001 patients could be collected retrospectively. In most cases, only one tumour bearing tissue block was available. Staging of tumours was accomplished using UICC TNM 7. Although only 49.7% of tumour samples of the CONKO-001 trial were collected, the data regarding clinical and histopathological features of the subset with available tumour tissue (Supplementary table 1) are comparable with the overall intention to treat population. There were no relevant differences between the subgroups.

### Quantification of tumour buds

About 2-μm-thick sections were cut from representative FFPE tissue blocks of 173 tumours of the CONKO-001 study and from 37 PDACs with resected synchronous liver metastasis, retrieved from the archives of the Institute of Pathology, Charité —Universitätsmedizin Berlin. The slides were stained with H&E, according to standard protocols. In most cases, only one tissue block of each tumour was available. In cases with more tumour-bearing tissue blocks, all blocks were cut and the one with the highest number of tumour buds was analysed. A tumour bud was defined as a single tumour cell or (non-glandular) clusters of up to four tumour cells.^5, 7^

To demonstrate the reproducibility of the tumour bud count, ten cases of PDAC were retrieved from the archives of the Institute of Pathology, Charité—Universitätsmedizin Berlin. From all cases, three slides from different tumour areas were chosen and five images at 400× magnification of representative areas of the tumour were taken from each slide, resulting in 150 images each with 952 × 768 pixels. The number of tumour buds on the images were independently counted by two observers, one an experienced pathologist (P.L.) and the other a non-pathologist trained to detect tumour buds (A.J.). The intra-class correlation coefficient between both observers was 0.904 (95% CI 0.729–0.959) for the total number of tumour buds per case.

Subsequently, counting of tumour buds was accomplished by an experienced pathologist (P.L.) blinded to clinical outcome using an Olympus BX40 microscope (Olympus Europe Holding GmbH, Hamburg, Germany).

**Fig. 1:**
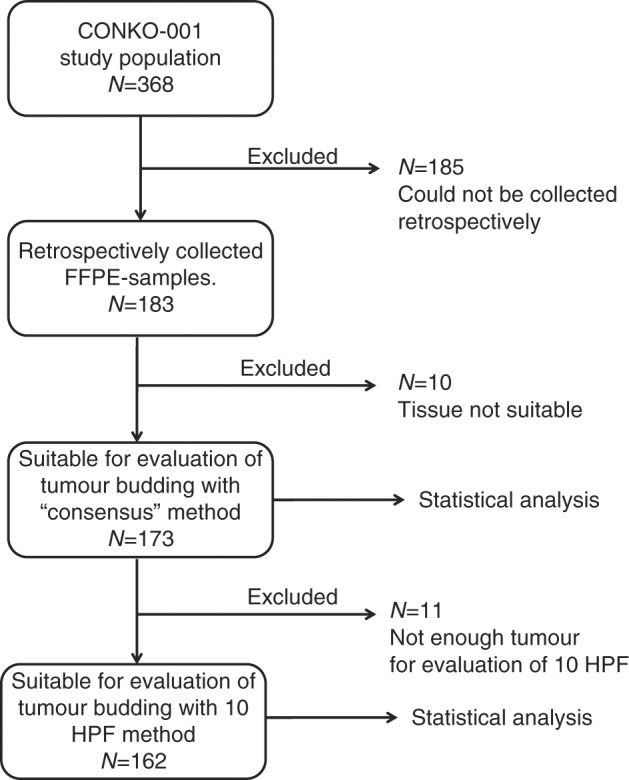
Study design. About 183 tissue blocks could be collected retrospectively from the original study population (*N* = 368). Of these tissue blocks, 173 were suitable for analysis with the consensus method. Another 11 samples had to be excluded for analysis with the 10 HPF method (*N* = 162)

Two different approaches to quantify the number of tumour buds were used (Fig. 1). (1) We used the quantification approach that was recently published by ITBCC^5^ for reporting tumour budding in colorectal cancer. Therefore, tumour buds were counted in one field of view (20× objective, 22 mm field of view ocular) at the “hotspot” of budding and the number of tumour buds per 0.785 mm^2^ was determined using a normalisation factor, as published.^5^ Budding was grouped according to ITBCC into Bd 1 (0–4 buds), Bd 2 (5–9 buds) and Bd 3 (10 or more buds). Using this method, 173 PDACs of the CONKO-001 study could be analysed. (2) Additionally, in areas of maximal tumour budding, detected at scanning magnification, the number of tumour buds was counted in 10 high-power fields (1 HPF 0.238 mm^2^, 40× objective; 22 mm field of view ocular). Since we, like others,^8, 9^ frequently observed tumour buds within the central parts of the tumour and because of difficulties in defining the edge of tumours, tumour budding was not subclassified into peritumoural and intra-tumoural. Using this method, 162 tumours were evaluable; 11 tumours had to be excluded, since less than 10 HPF could be analysed.

### Statistical considerations

DFS was defined as time from study entry to local or distant disease relapse or death, whichever came first. OS was defined as time from study entry to death due to any cause.

To investigate the prognostic impact of tumour buds for both quantification approaches, Cox regression analyses without a cut-off, using continuous data and proportional hazards regressions, were performed. Proportional hazards regression was calculated for multivariable and univariable survival models. The potential relevant clinical variables tumour budding, treatment arm, age, sex, pT stage, pN stage, grading and resection margin status were subsequently included in a multivariable proportional hazards model.

The Kaplan–Meier method with log-rank tests was used for univariable survival analyses. Cut-offs were as published for colorectal cancer,^5^ with only slight modifications. Pearsons correlation method was used to correlate tumour budding groups and clinicopathological parameters. Student's *T*-test was used for statistical analysis of the differences in the mean of tumour buds between groups with and without metastasis of the CONKO-001 cohort and the 38 PDACs with synchronous liver metastasis. In general, *p* values were calculated 2-sided and considered as significant when <0.05.

The Reporting Recommendations for Tumor Marker Prognostic Studies (REMARK) criteria were followed for reporting this study.^10^

## Results

### Correlation of tumour budding with clinicopathological parameters

**Fig. 2:**
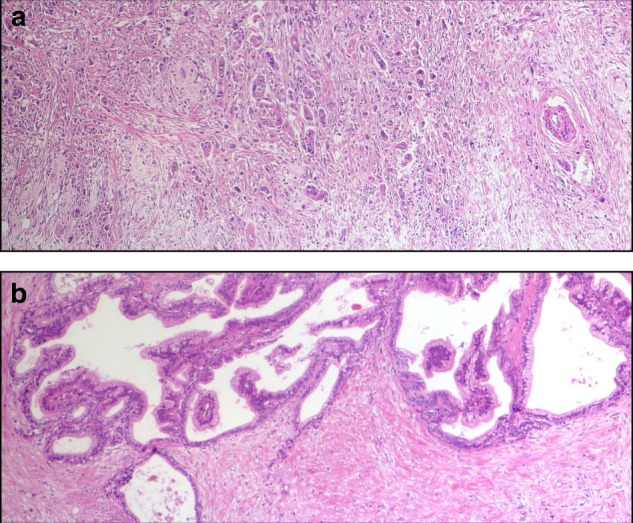
**a** Representative case with a high amount of tumour buds (magnification ×400). **b** Representative case with a low amount of tumour buds (magnification ×400)

With the quantification approach, recently published by the ITBCC,^5^ all 173 tumours were evaluable. The mean number of tumour buds was 8.0, the median 5.8 (range 0–35). Eighty-three tumours had 0–4 buds (Bd 1) per 0.785 mm^2^, 33 tumours had 5–9 buds (Bd 2) per 0.785 mm^2^ and 57 tumours could be assigned to Bd 3 with more than 9 buds per 0.785 mm^2^. When Bd 1 and 2 were subsumed in the low budding group, this group comprised 116 patients, the low budding group (Bd 3) 57 patients; 162 tumours could be analysed with the 10 HPF approach. The mean number of tumour buds per HPF was 5, the median 3.1 (range 0–23). Representative cases with high and low numbers of tumour buds are shown in Fig. 2.

We assessed the correlation of the clinicopathological parameters, patients age (<65 and ≥65 years), sex (male and female), Karnofsky Performance Status Scale Score (≤80 and >80), pT classification (pT1/2 and pT3/4), nodal status (pN0 and pN1), resection margins (R0 and R1), and grading (G1/2 and G3), with the budding groups (low budding and high budding) (Table 1). We found a highly significant positive correlation (*p* < 0.001) between the budding groups and tumour grade.

**Table 1:** Clinicopathologic correlation with low and high tumour budding

	Low budding *N* (%)	High budding *N* (%)	*p*
*Age*			
<65 years	77 (66)	33 (58)	0.276
≥65 years	39 (34)	24 (42)	
*Sex*			
Male	52 (45)	19 (33)	0.149
Female	64 (55)	38 (67)	
*Karnofsky Performance Status Scale Score*			
≤80	47 (45)	22 (48)	0.765
>80	57 (55)	24 (52)	
*pT stage*			
pT1/2	12 (10)	8 (14)	0.476
pT3/4	104 (90)	49 (86)	
*pN stage*			
pN0	28 (24)	14 (25)	0.951
pN1	88 (76)	43 (75)	
*Grading*			
G1/2	81 (73)	15 (27)	<0.001
G3	33 (27)	41 (73)	

When analysing the mean number of tumour buds, determined with the 10 HPF method, high-grade tumours had significantly more tumour buds than low-grade tumours. G1/2 tumours had a mean number of 2.74 tumour buds per HPF and G3 tumours 7.99 tumour buds per HPF (*p* < 0.001).

### A high number of tumour buds is associated with shorter DFS and OS

Recently a consensus method for determining the number of tumour buds in colorectal cancer^5^ recommended to determine the number of tumour buds in only one 0.785 mm^2^ field of view with maximal tumour budding. We tested if this method is also able to discriminate PDACs with a dismal from those with a better prognosis.

We first investigated the prognostic impact of tumour buds by performing a Cox regression analysis without a cut-off, using continuous data (Table 2). All 173 tumours were evaluable. An increased number of tumour buds was a significant negative prognostic marker for DFS and OS in all studied patients (DFS: HR = 1.037 (95% CI 1.017–1.058), *p* < 0.001; OS: HR = 1.040 (95% CI 1.019–1.061), *p* < 0.001). When stratifying into treatment arms, this effect was present in patients treated with adjuvant gemcitabine (DFS: HR = 1.042 (95% CI 1.015–1.069), *p* = 0.002; OS: HR = 1.040 (95% CI 1.014–1.067), *p* = 0.002) and in the untreated control group (DFS: HR = 1.041 (95% CI 1.006–1.077), *p* = 0.02; OS: HR = 1.038 (95% CI 1.003–1.075), *p* = 0.033).

**Table 2:** Survival in dependence of tumour budding

	Consensus method			10 HPF method		
	Risk ratio	95% Confidence interval	*p*	Risk ratio	95% Confidence interval	*p*
DFS all patients	1.04	1.02–1.06	<0.001	1.06	1.02–1.09	0.001
DFS treatment arm	1.04	1.02–1.07	0.002	1.07	1.02–1.12	0.005
DFS observation arm	1.04	1.01–1.08	0.002	1.04	0.99–1.09	0.12
OS all patients	1.04	1.02–1.06	<0.001	1.05	1.02–1.09	0.002
OS treatment arm	1.04	1.01–1.07	0.002	1.07	1.02–1.12	0.006
OS observation arm	1.04	1.00–1.08	0.033	1.04	1.00–1.09	0.11

**Fig. 3:**
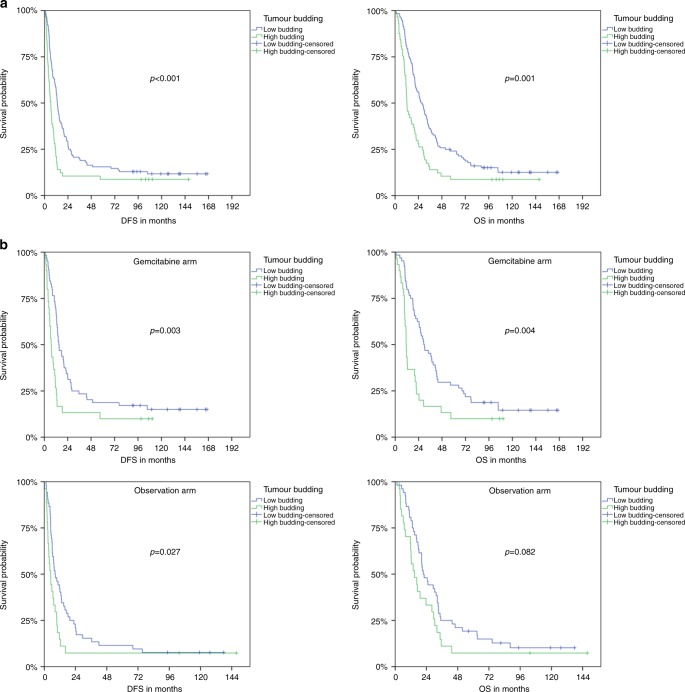
**a** Kaplan–Meier plots of median disease-free survival and overall survival for the overall CONKO-001 study group in dependence of a high and low tumour budding. **b** Kaplan–Meier plots of median disease-free survival and overall survival for the gemcitabine group and observation group of the CONKO-001 trial in dependence of high and low tumour budding

We next assigned the tumours to the three budding groups Bd 1 (*N* = 83), Bd 2 (*N* = 33) and Bd 3 (*N* = 57). Since there was no significant difference between Bd 1 and 2 (median DFS/OS 13.21/28.03 and 13.31/25.20, *p* = 0.484/0.527), we subsumed Bd 1 and 2 in the low budding group, while Bd 3 was referred to as high budding group. Low budding tumours were significantly associated with a longer DFS and OS in the overall study population. Median DFS/OS were 13.1/26.7 months for low budding tumours and 6.4/12.3 months for high budding tumours (*p* < 0.001/ = 0.001) (Fig. 3a).

In the subgroup analysis, significant differences were found in DFS and OS of gemcitabine-treated patients, as well as in DFS of patients in the observation group. In the gemcitabine group, median DFS/OS was 15.0/29.2 months for low budding tumours and 7.0/11.2 months for high budding tumours (*p* = 0.003/0.004). In the observation group, patients of the low budding group had a median DFS/OS of 8.3/21.5 months compared to 4.9/14.9 months for patients of the high budding group (*p* = 0.027/0.082) (Fig. 3b).

Additionally a multivariable survival analysis, including budding group, treatment arm, age, sex, pT stage, pN stage, grading and resection margin status (Table 3). Only pT stage (HR = 2.393 (95% CI 1.342–4.268), *p* = 0.003), budding groups (HR = 2.222 (95% CI 1.460–3.383), *p* < 0.001), treatment arm (HR = 1.669 (95% CI 1.207–2.308), *p* = 0.002) and pN stage (HR = 1.633 (95% CI 1.100–2.422), *p* = 0.015) retained their prognostic status on DFS. The pT stage (HR = 2.402 (95% CI 1.355–4.259), *p* = 0.003), budding groups (HR = 1.935 (95% CI 1.294–2.893), *p* = 0.001), pN stage (HR = 1.612 (95% CI 1.084–2.398), *p* = 0.018) and grading (HR = 1.589 (95% CI 1.111–2.273), *p* = 0.011) retained their prognostic status on OS.

**Table 3:** Multivariable survival analysis

	DFS			OS		
Variable	Risk ratio	95% Confidence interval	*p*	Risk ratio	95% Confidence interval	*p*
Tumour budding (high vs. low)	2.22	1.46–3.38	<0.001	1.94	1.29–2.89	0.001
Treatment arm (Gem vs. Obs)	1.67	1.21–2.31	0.002	1.15	0.83–1.60	0.394
pT (pT1/2 vs. 3/4)	2.39	1.34–4.27	0.003	2.40	1.36–4.26	0.003
pN (pN0 vs. pN1)	1.63	1.10–2.42	0.015	1.61	1.08–2.40	0.018
Grading (G1/2 vs. G3/4)	1.32	0.92–1.90	0.135	1.59	1.11–2.27	0.011
Resection margin (R0 vs. R1)	1.45	0.94–2.24	0.095	1.20	0.77–1.86	0.414
Age (≤65 y vs. >65 y)	0.91	0.65–1.29	0.600	0.87	0.62–1.23	0.442
Sex (m vs. f)	1.204	0.86–1.69	0.286	1.22	0.87–1.72	0.254

We next investigated the prognostic impact of tumour buds by performing a Cox regression analysis without a cut-off, using continuous data of 162 tumours analysed with the 10 HPF approach (Table 2). An increased number of tumour buds was a significant negative prognostic marker for DFS and OS in all studied patients (DFS: HR = 1.056 (95% CI 1.022–1.092), *p* = 0.001; OS: HR = 1.052 (95% CI 1.018–1.087), *p* = 0.002). When stratifying into treatment arms, this effect was only detectable in patients treated with adjuvant gemcitabine (DFS: HR = 1.071 (95% CI 1.021–1.123), *p* = 0.005; OS: HR = 1.069 (95% CI 1.019–1.121), *p* = 0.006) but not in the untreated control group (DFS: HR = 1.038 (95% CI 0.990–1.088), *p* = 0.12; OS: HR = 1.038 (95% CI 0.991–1.087), *p* = 0.112). The test for interaction was not significant (*p* = 0.474).

### Association of tumour buds with distant metastasis

Tumour budding is regarded as a morphologic detectable sign of EMT and EMT is seen as a crucial step in the development of metastasis. We therefore asked whether a higher number of tumour buds is associated with the development of distant metastasis in PDAC of the CONKO-001 trial. However, there was no correlation between the budding group and the presence of distant metastases and no significant difference between the number of tumour buds per 10 HPF in PDACs with and without distant metastasis (mean number 4.93 (95% CI 3.88–5.99) and 3.68 (95% CI 1.75–5.61), respectively; *p* = 0.262).

We additionally analysed for the first time the number of tumour buds in a set of 38 resected PDACs with resected and histologically proven synchronous liver metastasis. The mean number of tumour buds was 4.24 (95% CI 2.72–5.76). Again there was no significant difference in the number of tumour buds between the cases with synchronous liver metastasis and the cases with (mean number 4.24 vs. 4.93; *p* = 0.672) or without (mean number 4.24 vs. 3.89; *p* = 0.658) distant metastasis of the CONKO-001 cohort.

## Discussion

In the present study, we retrospectively evaluated the prognostic effect of tumour budding on PDACs of the CONKO-001 study, encompassing two different groups: one treated with adjuvant gemcitabine and an untreated control group. Tumour budding is best described as a certain type of invasive growth pattern which can be observed at histological examination of carcinomas. Tumour buds are defined as single cells or non-glandular clusters of less than five cells.^7^ Tumour budding has been extensively studied in colorectal carcinoma,^7, 11–14^ but has also been described in various other adenocarcinomas like ampullary cancer,^15^ breast cancer,^16^ lung cancer,^17^ gastric cancer,^18, 19^ oesophageal cancer and cancer of the gastro-oesophageal junction^20^ and in squamous cell carcinomas like oesophageal squamous cell carcinomas.^21, 22^ In all studied tumour entities, the presence of tumour budding was associated with a worse prognosis. In pancreatic cancer, there are only limited studies on retrospectively collected tumour cohorts using different cut-offs and quantification methods, that found tumour budding to be an adverse prognostic factor.^8, 9, 23–25^

Using H&E-stained sections from FFPE tissue from patients of the prospective CONKO-001 trial, we could demonstrate that the presence of tumour buds are associated with highly significant differences on patient outcome. Shortly, two different approaches to quantify tumour buds were used. We used the quantification approach that has recently been included in a standardised evidence-based consensus scoring system for tumour budding in colorectal cancer.^5^ Additionally, we determined the mean number of tumour buds in 10 HPF. Cut-off free Cox regression analyses using continuous data of both quantification methods, without a cut-off, showed that the patients with high numbers of tumour buds had a significantly worse DFS and OS, compared to those with lower numbers. After stratification into an observation arm and a treatment arm, there were no significant differences in DFS, however concerning OS  the prognostic effect was restricted to patients in the treatment arm (i.e., adjuvant gemcitabine). In addition, we demonstrated that PDAC could be stratified into prognostic subgroups by using the proposed budding groups with only slight modification. We, like others,^9^ found a highly significant positive correlation between the number of tumour buds and tumour grading. In our study, a higher number of tumour buds was significantly correlated with poorly differentiated tumours. This might at least in part be due to some overlapping between tumour grade and tumour budding. We did not subclassify into peritumoural and intra-tumoural budding because of difficulties in defining the edge of the tumours and the frequent observation of intra-tumoural buds in PDAC.^8, 9^ Grading of PDAC according to the WHO^26^ includes the semi-quantitative assessment of mucin production, mitoses, nuclear atypia and glandular differentiation.^27^ Poorly differentiated tumours contain poorly formed glands and can show individual infiltrating cells and solid areas. This might at least in part explain our here-found association between grading and tumour budding. However, not all poorly differentiated tumours show high budding, and in multivariate analysis, tumour budding was an independent prognostic factor. Together with previously published data,^8, 9, 23–25^ our results link tumour budding to a worse prognosis in PDAC and underline the aggressive nature of budding-positive tumours.

Both quantification methods are able to discriminate prognostic subgroups in patients adjuvantly treated with gemcitabine. However, when directly comparing both quantification methods, the consensus method might be the method of choice for daily practice for several reasons. It is the same quantification approach as already known from colorectal carcinoma and as only one field of view has to be counted, it is not as time consuming as the 10 HPF approach. Moreover, this method was also able to discriminate prognostic subgroups in patients with PDAC in the observation arm of CONKO 001.

Tumour budding is discussed to represent the morphological correlate of EMT. The loss of epithelial characteristics and gain of mesenchymal characteristics reduces cell adhesion and enhances cell motility. Therefore, EMT is seen as a first step towards metastasis formation. Indeed, tumour budding has been linked to the presence of distant metastases in colorectal cancer.^28, 29^ However, in our investigations in PDAC, we found no significant difference in the number of tumour buds between cases from the CONKO-001 trial with metachronous distant metastasis and no distant metastasis. We additionally determined from the mean number of tumour buds in 10 HPF from 38 PDACs with resected and histologically proven synchronous liver metastasis. We compared the mean number of tumour buds to PDAC of the CONKO-001 trial with and without distant metastasis and found no significant differences between the groups. We suppose that this might be due to different reasons: the fact that tumour budding is more correct to be regarded a “partial” EMT,^30^ the quite complex orchestrated mechanisms of metastasis induction and the aggressive nature of PDAC with a comparatively short survival. In PDAC, immunohistochemical studies detected a reduced, yet not abolished, E-cadherin expression and nuclear beta catenin expression in tumour buds.^23^ In other studies nuclear beta catenin expression could not be demonstrated in PDAC, indicating that the EMT-inducing activation of the canonical Wnt pathway is not a prerequisite for tumour budding in PDAC.^31^ Although tumour buds appear disconnected from the main tumour mass in two-dimensional (2D) histological slides, studies on 3D reconstructions and serial section showed that tumour buds are actually connected to the main tumour mass.^32^ These findings imply that there is no complete transition to a mesenchymal phenotype, therefore tumour budding is discussed to be “partial” EMT. After the induction of EMT, metastatic cancer cells migrate away from the original tumour, and spread to distant sites.^33^ There a mesenchymal–epithelial transformation (MET) is required for successful development of metastasis. According to the “seed and soil” hypothesis by Paget,^34^ the microenvironment of the distant site is crucial for the development of distant metastasis. Taken together, EMT is only the first step of the highly complex process of metastasis formation, and tumour budding might not completely reflect EMT. This might at least partially explain why in our study the presence of tumour buds in PDAC did not correlate with metastasis formation.

Our study has limitations. For our here-presented analysis, 183 tissue samples of the initial 354 CONKO-001 patients were available and due to low quality of tissue fixation or limited tumour in the paraffin blocks, only 173 samples were suitable for analysis of tumour buds. Although only 173 (47%) tumour samples of the CONKO-001 trial were retrospectively collected and eligible for further analysis, the data regarding clinical and histopathological features of the subset with available tumour tissue are comparable with the overall intention to treat the population. Another limitation may be the fact that in most cases only one tumour-bearing tissue block was available. We used H&E-stained slides to determine the number of tumour buds, which—when compared to enumeration with the use of cytokeratin stains—could underestimate the real number of tumour buds.^35^ Although the use of cytokeratin stains has been described to be useful in identifying tumour buds, we used H&E-stained slides for several reasons. First, the quantification of tumour buds on H&E-stained slides can be easily implemented in the daily routine of surgical pathologists. Second, we and others^9^ have demonstrated a good reproducibility in recognising tumour buds in H&E-stained sections of PDAC. Third, the quantification of tumour buds in H&E-stained slides is consensus in colorectal cancer.^5^

To summarise, we show data on the impact of tumour buds in resected PDACs using two different quantification methods. We could demonstrate a positive correlation of tumour budding and tumour grade. Further, high numbers of tumour buds were associated with a significantly worse survival in resected PDACs. In contrast to previous published data in colorectal cancer, we could not demonstrate that tumour budding in PDAC is associated with metastasis formation. Analysis of tumour buds in PDAC could be easily implemented in routine diagnostic processes, but needs further standardisation and verification by prospective studies.

## Electronic supplementary material

Supplementary Table 1

## Availability of data and material

The data supporting the conclusions of this article are stored in the Charité–Universitätsmedizin Berlin, Institute of Pathology.
