# Supplementary material for: Tumour buds determine prognosis in resected pancreatic ductal adenocarcinoma
Source: Br J Cancer. 2018 May 14;118(11):1485–91. doi: 10.1038/s41416-018-0093-y (PMC5988658; doi:10.1038/s41416-018-0093-y)
Supplement: Supplementary file 1 — Supplementary Table 1 [file 41416_2018_93_MOESM1_ESM.docx]

| **Age** |  |  |
| --- | --- | --- |
| median | 62 |  |
| range | 36-81 |  |
| **Survival (months)** |  |  |
| Median DFS (95% CI) | 10.6 | (8.1-13.0) |
| Median OS (95% CI) | 21.5 | (17.9-25.0) |
| **Karnofsky Performance Status Scale Score** |  |  |
| median | 80 |  |
| range | 50-100 |  |
| N=173 | **N** | **%** |
| **Treatment Arm** |  |  |
| Gemcitabine | 94 | 54.3 |
| Observation | 79 | 45.7 |
| **Gender** |  |  |
| Female | 71 | 41.0 |
| Male | 102 | 59.0 |
| **T stage** |  |  |
| pT1–2 | 20 | 11.6 |
| pT3–4 | 153 | 88.4 |
| **Nodal status** |  |  |
| pN0 | 42 | 24.3 |
| pN1 | 131 | 75.7 |
| **Grading** |  |  |
| G1–2 | 98 | 56.6 |
| G3 | 75 | 43.4 |
| **Resection margin** |  |  |
| R0 | 143 | 82.7 |
| R1 | 30 | 17.3 |

|  |
| --- |
